# From the Thoracic Outlet to the Brachial Plexus: A Rare Presentation of Pediatric Ewing Sarcoma

**DOI:** 10.7759/cureus.116315

**Published:** 2026-09-15

**Authors:** Mahy Mohamed El Kerim, Manal Azizi, Amal Bennani, Imane Kamaoui, Ayad Ghanam

**Affiliations:** 1 Pediatrics, Mohammed VI University Hospital, Oujda, MAR; 2 Pediatrics, Faculty of Medicine and Pharmacy, Mohammed VI University Hospital, Mohammed 1st University, Oujda, MAR; 3 Anatomopathology, Faculty of Medicine and Pharmacy, Mohammed VI University Hospital, Mohammed 1st University, Oujda, MAR; 4 Oncoradiology, Faculty of Medicine and Pharmacy, Mohammed VI University Hospital, Mohammed 1st University, Oujda, MAR

**Keywords:** brachial plexus, ewing sarcoma, first rib, pediatric oncology, thoracic outlet syndrome

## Abstract

Ewing sarcoma of the chest wall (CWES) is a highly aggressive mesenchymal tumor, the incidence of which in children under five years of age remains extremely rare. Primary involvement of the first rib and the apex of the thorax presents specific anatomical challenges, leading to severe compressive complications at an early stage. We report the case of a four-year-and-two-month-old girl admitted for right-sided neuropathic arm pain that had been present for three months and was initially attributed to a minor trauma. The gradual onset of upper extremity muscle atrophy, motor deficit predominantly affecting the intrinsic muscles of the hand, supraclavicular swelling, and a nocturnal febrile syndrome prompted a cross-sectional imaging workup. A cervicothoracoabdominopelvic CT scan revealed a large necrotic mass in the right cervicothoracic region, arising from the first rib and compressing the trunks of the brachial plexus and the subclavian artery, causing collapse of the innominate venous trunk and intraspinal extension from C7 to T1. An ultrasound-guided percutaneous biopsy established the diagnosis of Ewing sarcoma (monomorphic round blue cells, CD99+, FLI-1+) and was genetically confirmed by detection of an EWSR1 gene rearrangement via fluorescence in situ hybridization (FISH). Systemic staging evaluation revealed no distant metastases. The onset of thoracic outlet syndrome in a very young child requires immediate consideration of a malignant apico-thoracic tumor as a possible cause. Multidisciplinary pediatric management combining early induction chemotherapy and an appropriate local control strategy is essential for achieving recurrence-free survival while minimizing irreversible functional sequelae.

## Introduction

Ewing sarcoma is an aggressive malignant mesenchymal neoplasm that primarily affects bone structures and soft tissues in children, adolescents, and young adults [1]. First described in 1921 by pathologist James Ewing, this tumor group belongs to the classic spectrum of 'small blue round-cell tumors' [2]. The Ewing tumor family includes bone Ewing sarcoma, extraosseous Ewing sarcoma, primitive neuroectodermal tumor (PNET), and Askin tumor (primitive Ewing sarcoma of the chest wall) [3]. These entities, which were once distinguished based on morphological criteria, are now grouped within a single nosological category following the identification of their common molecular signature [4]. Virtually all of these tumors are characterized by a recurrent chromosomal translocation involving the EWSR1 gene located on chromosome 22q12, most often resulting in the chimeric EWSR1-FLI1 fusion gene, which has significant oncogenic activity [5].

Epidemiologically, Ewing sarcoma is the second most common malignant bone tumor in children after osteosarcoma, with peak incidence observed between the ages of 10 and 15 [6]. Primary chest wall Ewing sarcomas (CWES) account for approximately 15% to 20% of all such cases, making them a rare entity in routine pediatric practice [7]. Clinically, local or regional pain is the most consistent presenting symptom; it is often intermittent, progressively worsening, and does not subside with nighttime rest [8]. This pain is usually followed by the appearance of a palpable mass, although pelvic, femoral, or thoracic lesions may remain asymptomatic for an extended period. Signs of vascular-nerve or spinal cord compression constitute major medical emergencies requiring immediate medical and surgical intervention [9]. The average time between the onset of initial symptoms and definitive diagnosis ranges from three to nine months in the literature [10]. In this context, we report the case of a four-year-and-two-month-old girl with Ewing sarcoma of the chest wall arising from the first rib, which was revealed by thoracic outlet syndrome and had a diagnostic delay of three months.

## Case presentation

The patient is a four-year-and-two-month-old girl with no significant personal or family medical history, admitted for evaluation of pain in her right upper limb that had been present for three months. This pain, which was clearly neuropathic in nature, worsened markedly at night and was accompanied by progressive functional impairment, as the girl was no longer able to grasp everyday objects. The symptoms had initially been attributed to a minor trauma and treated as such with first-line analgesics, with no improvement. The course of the illness was marked by persistent arm pain associated with tingling-type paresthesias, progressive muscle wasting in the affected limb, and the recent appearance of a right supraclavicular swelling, against a background of deteriorating general health characterized by prolonged nocturnal fever, measurable weight loss, anorexia, and severe asthenia. 

The clinical examination upon admission revealed a girl who was conscious, oriented, and hemodynamically and respiratorily stable but presenting with severe hyperalgesia rated at 6/10 on the visual analog scale (VAS) and a fever of 38.9°C. The musculoskeletal examination revealed a characteristic antalgic posture of the right upper limb, atrophy of the right forearm compared to the contralateral side, and sharp pain upon palpation of its anteromedial aspect. Segmental neurological examination revealed a motor deficit on the affected side of 4/5 in the proximal muscles of the arm and 3/5 in the intrinsic muscles of the right hand, associated with a general decrease in osteotendinous reflexes of the right upper limb (biceps, triceps, and styloradial reflexes). Examination of the cervical and thoracic regions revealed a mass in the right supraclavicular fossa measuring 3 cm in its longest axis, firm in consistency, fixed to the deep planes, and tender to pressure, with no obvious signs of superior vena cava syndrome. Pleuropulmonary auscultation revealed diminished vesicular breath sounds at the right pulmonary apex.

The initial laboratory workup revealed a systemic inflammatory response with elevated C-reactive protein (CRP) at 42.88 mg/L, contrasting with a normal leukocyte count of 7,800/mm³ (48.2% neutrophils, 42.8% lymphocytes). Renal and hepatic function were strictly normal, and serological tests for hepatitis B and C were negative (Table 1). A cervicothoracoabdominopelvic CT scan revealed a large, necrotic, heterogeneous tissue mass in the right cervical-thoracic region, measuring 80 × 73 × 70 mm. This lesion was accompanied by aggressive osteolysis of the middle arc of the first right rib, transforaminal and intraspinal extension at C7-T1 without signs of overt spinal cord compression, complete encasement of the trunks of the right brachial plexus, compression of the subclavian artery (which remained patent), and collapse of the right innominate venous trunk. There was also adjacent pulmonary parenchymal involvement leading to passive atelectasis of the right upper lobe (Figure 1).

**Table 1:** Initial laboratory workup CRP: C-reactive protein

Parameter		Value	Reference range	Interpretation
Complete blood count	White blood cells	7,800/mm³	4,000-10,000/mm³	Normal
	Red blood cells	4.34 M/mm³	4.0-5.2 M/mm³	Normal
	Hemoglobin	12.0 g/dL	11.5-15.5 g/dL	Normal
	Hematocrit	34.6%	34%-40%	Normal
	Platelets	345,000/mm³	150,000-400,000/mm³	Normal
Biochemistry	CRP	42.88 mg/L	< 5 mg/L	Elevated
	Lactate dehydrogenase	130 IU/L	120-300 IU/L (child)	Normal
	Aspartate aminotransferase (serum glutamic-oxaloacetic transaminase)	25 U/L	10-40 U/L (child)	Normal
	Alanine aminotransferase (serum glutamic-pyruvic transaminase)	13 U/L	10-40 U/L (child)	Normal
	Blood urea	0.23 g/L	0.15-0.45 g/L	Normal
	Creatinine	4.53 mg/L	3-7 mg/L (child)	Normal
	Uric acid	34.90 mg/L	20-60 mg/L	Normal
	Phosphorus	51.0 mg/L	40-65 mg/L (child)	Normal
	Calcium	99 mg/L	85-105 mg/L	Normal
	Sodium	136 mEq/L	135-145 mEq/L	Normal
	Potassium	4.2 mEq/L	3.5-5.1 mEq/L	Normal
	Total protein	75 g/L	60-80 g/L	Normal

**Figure 1:**
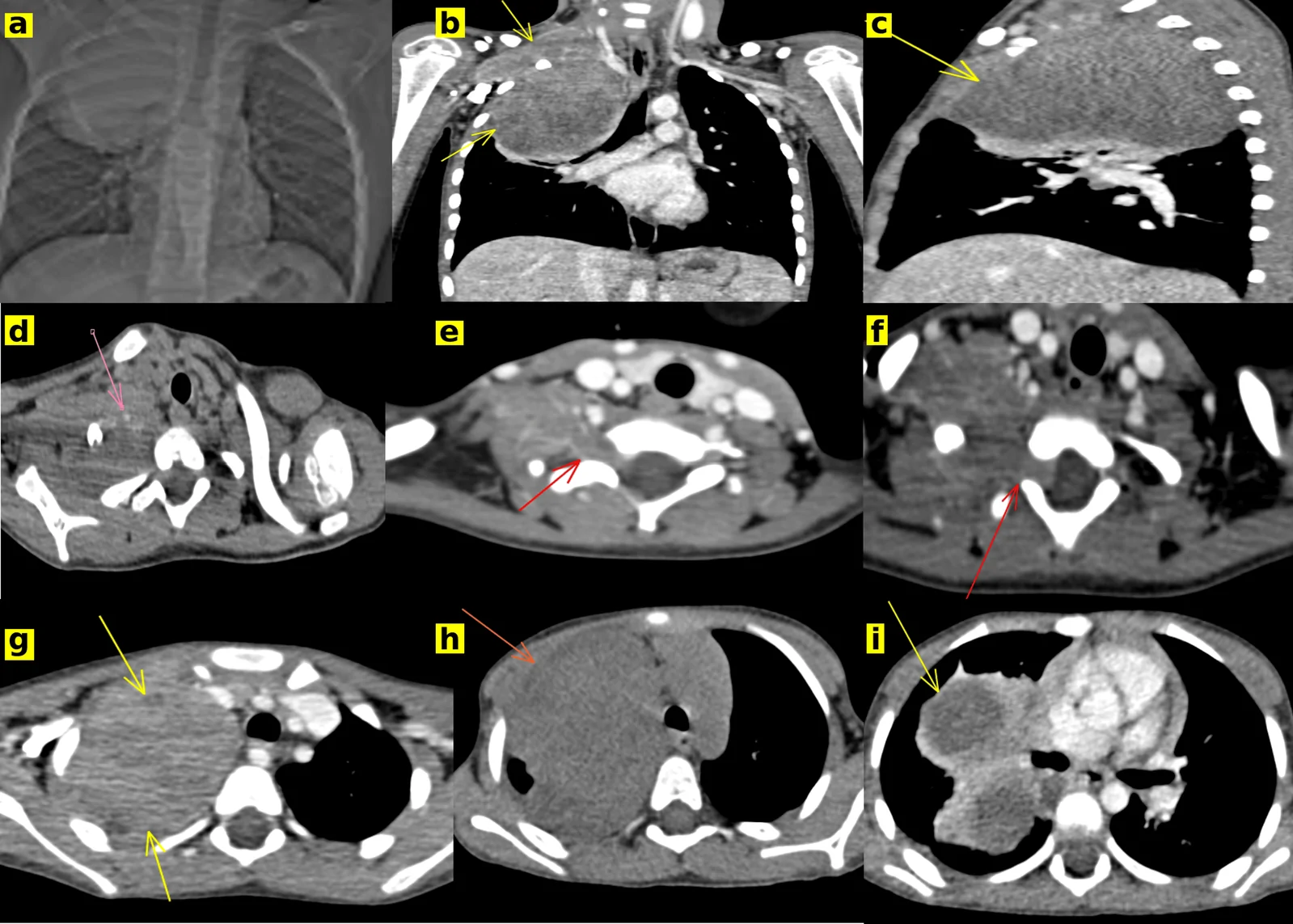
Axial, coronal, and sagittal CT images showing a large necrotic mass in the cervicothoracic region, arising from the first rib a: Coronal bone-window reconstruction showing osteolysis of the first rib; b: Coronal view of the mass extending into the supraclavicular and cervicothoracic region (yellow arrows); c: Sagittal view showing the craniocaudal extent of the mass along the chest wall (yellow arrow); d: Axial view of intratumoral microcalcifications (pink arrow); e and f: Axial view of tumor extension into the intervertebral foramen and spinal canal at C7–T1 (red arrows); g: Axial view of the tumor encasing the trunks of the brachial plexus (yellow arrows); h: Axial view of anterior extension of the mass toward the chest wall (orange arrow); i: Axial view of compression of the subclavian artery and collapse of the innominate venous trunk (yellow arrow)

Ultrasound-guided percutaneous biopsy revealed, upon histopathological examination, a diffuse tumor proliferation composed of small, round, monomorphic blue cells with hyperchromatic nuclei and scant cytoplasm. Immunohistochemical analysis showed intense, diffuse membrane expression of CD99 (MIC2) as well as positivity for the FLI-1 transcription factor, with no staining for chromogranin, synaptophysin, CD20, CD3, desmin, myogenin, or pancytokeratin (Figure 2). Genetic confirmation was provided by fluorescence in situ hybridization (FISH), which demonstrated the presence of an EWSR1 gene rearrangement at the 22q12 locus. Systemic staging, including a technetium-99m bone scan, a bilateral bone marrow aspirate, and bilateral bone marrow biopsy (BMB), revealed no secondary bone or bone marrow metastases. The diagnosis was locally advanced cervicothoracic Ewing sarcoma, arising from the first rib and presenting as thoracic outlet syndrome.

**Figure 2:**
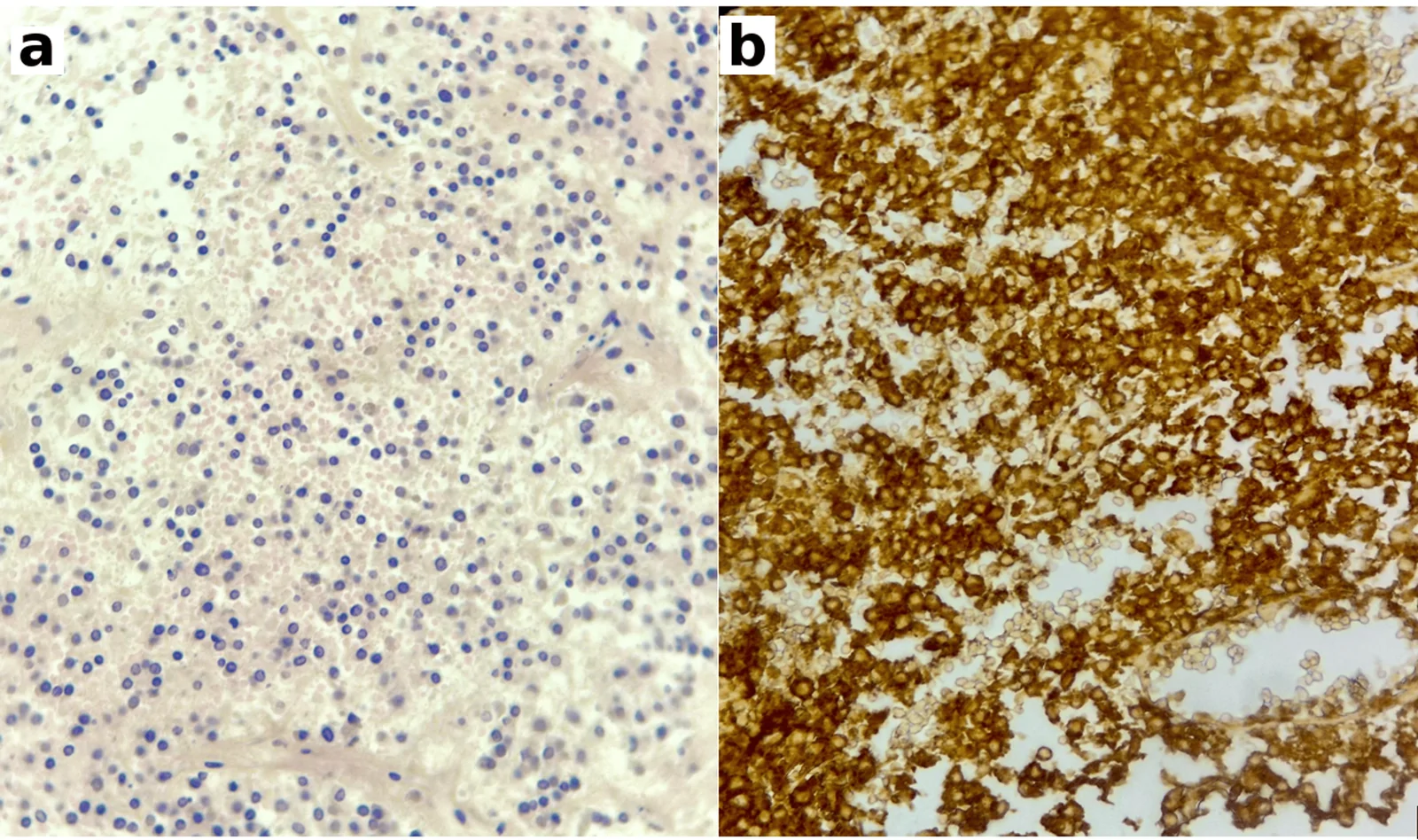
Immunohistochemical analysis a: Diffuse proliferation of monomorphic small round cells with hyperchromatic nuclei and scant cytoplasm (H&E stain, ×40);  b: Diffuse, intense membranous CD99 expression by tumor cells (immunohistochemistry (IHC) ×40)

## Discussion

Ewing sarcoma is the second most common malignant bone tumor in children, but its occurrence in children under five years of age is extremely rare [1,2]. Fewer than 5% of cases recorded in major international cancer registries, such as the North American Surveillance, Epidemiology, and End Results (SEER) registry, occur in this early age group [3]. Although tumors involving the chest wall account for approximately 15% to 20% of all Ewing sarcomas, primary involvement of the first rib occurs in fewer than 2% of thoracic cases [4,5]. The combination, in an infant or very young child, of an upper rib origin and tumor growth within the cervicothoracic outlet makes this case exceptionally rare. Anatomically, the first rib forms the bony framework of a narrow passage through which the major vascular and nervous structures supplying the upper limb travel [6]. Unlike in the middle or lower rib segments, where the tumor expands freely toward the pleural cavity as a mass that becomes symptomatic only later, tumor growth at the thoracic apex early on constricts the interscalene triangle and the costoclavicular space, transforming an emerging neoplastic process into an acute anatomical conflict [7].

This particular topographical configuration explains the atypical clinical presentation of thoracic outlet syndrome. Thoracic outlet syndrome is rare in young children, a population in which it is almost exclusively caused by congenital structural anomalies such as a cervical rib or a fibrous band [8]. Its manifestation as a primary malignant tumor frequently leads to initial misdiagnosis. In our patient, the initial attribution of the pain to a benign microtrauma reflects a classic clinical anchoring bias. This three-month diagnostic delay was exacerbated by the initial absence of a visible mass in the supraclavicular fossa and by the misleading nature of the neurological findings. The involvement of the lower primary trunk of the brachial plexus (C8-T1) caused nocturnal radicular pain, paresthesias, and selective weakness of the intrinsic muscles of the hand, resulting in a picture of Dejerine-Klumpke paralysis [10]. It was only at the stage of significant volumetric expansion that the mass became palpable, accompanied by vascular compromise due to compression of the subclavian artery and collapse of the innominate venous trunk [11].

The presence of marked deterioration in the patient’s general condition, combined with a nocturnal fever and an isolated elevation of C-reactive protein at 42.88 mg/L, contrasting with a normal leukocyte count, presented an additional diagnostic challenge. In pediatric practice, the combination of prolonged fever, a biological inflammatory syndrome, and limb pain often suggests an infectious condition such as osteomyelitis of the first rib, septic sternoclavicular arthritis, or a soft-tissue abscess. Physiologically, this systemic reaction is secondary to the paracrinally-secreted pro-inflammatory cytokines, particularly interleukin-6 (IL-6) and TNF-alpha, by the tumor stroma and neoplastic cells [12,13]. The combination of a pseudo-infectious febrile presentation and thoracic outlet syndrome in a young child should therefore raise suspicion of an aggressive neoplastic proliferation in the cervicothoracic region.

From a pathological perspective, the detection of a proliferation of small, round, blue cells in a four-year-old child in the supraclavicular region necessitates a rigorous differential diagnosis. The main diagnoses to rule out include cervicothoracic neuroblastoma, embryonal rhabdomyosarcoma, and T- or B-cell lymphoma. Strong immunohistochemical positivity for CD99 (MIC2) and the FLI-1 transcription factor strongly suggests the Ewing tumor family, but the specificity of CD99 remains imperfect. The decisive step relies on molecular cytogenetics via FISH, which provides evidence of a break in the EWSR1 gene at the 22q12 locus [14]. In our patient, immunohistochemical analysis revealed CD99 expression and a positive result for the FLI-1 transcription factor, with no staining for chromogranin, synaptophysin, CD20, CD3, desmin, myogenin, or pancytokeratin. The FISH technique demonstrated the presence of a rearrangement of the EWSR1 gene at the 22q12 locus, which confirmed the diagnosis of Ewing sarcoma and ruled out differential diagnoses such as neuroblastoma, rhabdomyosarcoma, and lymphoma.

The treatment of locally advanced cervicothoracic Ewing sarcoma involving the neurovascular structures of the thoracic outlet and extending into the spinal canal presents a therapeutic dilemma of great complexity [15]. Standard management relies on the immediate administration of intensive multi-drug induction chemotherapy combining vincristine, doxorubicin, cyclophosphamide, ifosfamide, and etoposide according to the international Euro-Ewing and Children’s Oncology Group protocols [16]. In cases involving the thoracic outlet, neoadjuvant chemotherapy aims not only to reduce the systemic micrometastatic tumor burden but also to achieve rapid local cytoreduction in order to relieve axial vascular and neurological compression [17]. The central challenge lies in choosing the modality for local control [18].

Although complete surgical resection with clear margins (R0) is the most powerful individual prognostic factor for recurrence-free survival in Ewing sarcoma of the chest wall, extended resection in the cervicothoracic region from the outset in a four-year-old child would result in the irreversible sacrifice of the brachial plexus trunks and permanent functional loss of the upper limb [19]. The recommended approach is to reevaluate the mass using MRI after administration of induction chemotherapy [20]. If tumor regression allows for the exposure of vascular and nerve structures, a conservative resection of the first rib with brachial plexus neurolysis should be prioritized [21]. In cases of persistent tumor involvement or when it is impossible to achieve R0 margins without major debridement, definitive radiation therapy combined with consolidation chemotherapy is the validated strategy for ensuring local tumor control while preserving neurological function of the upper limb [22]. In our case, the multidisciplinary team decided to initiate neoadjuvant chemotherapy according to the Euro-Ewing 99 protocol, followed by a radiological evaluation after the third cycle. The patient has already received two cycles, resulting in clinical improvement, notably the resolution of fever and regression of thoracic outlet syndrome.

## Conclusions

Ewing sarcoma of the first rib is an exceptionally rare condition in children under five years of age. Its presentation as thoracic outlet syndrome poses a major diagnostic challenge, as it is too often misdiagnosed as traumatic or infectious in origin. In any case of painful monoplegia or radicular pain in the upper limb associated with an inflammatory syndrome in a young child, prompt cross-sectional imaging of the upper thoracic aperture is essential. Only early identification of the lesion, molecular genetic confirmation of the EWSR1 translocation, and coordinated multidisciplinary pediatric care can reconcile the requirements of R0 oncological control with the preservation of vascular and nerve function in the upper limb.
